# Effects of Dental Occlusion on Body Sway, Upper Body Muscle Activity and Shooting Performance in Pistol Shooters

**DOI:** 10.1155/2018/9360103

**Published:** 2018-07-24

**Authors:** Amândio A. Dias, Luís A. Redinha, Luís M. Silva, Pedro C. Pezarat-Correia

**Affiliations:** ^1^Neuromuscular Research Lab, CIPER, Faculdade de Motricidade Humana, Universidade de Lisboa, 1495-687 Cruz Quebrada, Portugal; ^2^Instituto Piaget, Campus de Almada, 2805-059 Almada, Portugal; ^3^Faculdade de Medicina Dentária, Universidade de Lisboa, 1600-277 Lisboa, Portugal; ^4^Escola Superior de Hotelaria e Turismo do Estoril, 2769-510 Estoril, Portugal

## Abstract

Occlusal splints, to some extent, have been related to reduced body sway in a static position and increased muscle activity in the upper limbs. However, how dental occlusion status affects sports performance remains unclear. Here, we investigated whether occlusal splints that reposition the temporomandibular joint (TMJ) influenced body posture, muscle activity, and performance in 10-meter pistol shooters. Thirteen national-level male shooters (age = 38.8 ± 10.9 yrs) were recruited for this study, and cleared of any cervical pathology. An occlusal splint (OS) and a placebo splint (PS) were fabricated for each of the subjects, with the mandibular and maxillary position verified by an expert dentist, with the aid of an adjustable articulator. Surface electromyography (EMG) was assessed in the upper limb that holds the pistol while the subjects were standing on a force platform. Subjects performed two series of 10 shots for each of the three experimental conditions (OS, PS, N (no splint)) in randomized order, with the mandible in a rest position. Results revealed similar centre of pressure (COP) parameters in all conditions, despite a reduction in the average oscillation area caused by the OS. There were also no significant differences in EMG activity between conditions in the five upper limb muscles monitored. Consistent with this, shooting performance was similar in all conditions, despite a reduction in shot dispersion in subjects using OS. Thus, changes in dental occlusion status induced by OS do not affect body posture, upper limb EMG muscle activity, or shot performance in healthy male pistol shooters.

## 1. Introduction

Human posture refers to the position of the body that maintains balance in static conditions, including the spatial relations between its anatomical segments. Posture involves constant adjustments to respond to continuous oscillations in the upright position [1]. These adjustments require muscle activation and are controlled by the central nervous system (CNS), which integrates a variety of sensory inputs (visual, vestibular, and proprioceptive) from a complex system of sensors [1].

Previous studies have suggested that dental occlusion status influences posture control [2–4]. Dental occlusion is the relationship between the maxillary (upper) and mandibular (lower) teeth when they approach each other during chewing or at rest [5]. It was proposed that changes in dental occlusion affect body posture via output signals transmitted by the trigeminal nerve, which is associated to mandibular proprioception [6]. The altered signal is then transmitted to the CNS, which in turn transfers it to the entire body system via spinal and autonomic nerves [7]. Moreover, changes in occlusion status induced by cotton rolls or an occlusion splint (OS) demonstrated that posture can be affected by manipulations of the temporomandibular joint (TMJ) [2, 8–11]. For instance, by using cotton rolls to change the mandibular position, Baldini et al. showed that dental occlusion has a significant influence in body sway [2]. Another report reached similar conclusions by using a force platform to measure the effects of three different mandibular positions on body posture [8]. However, some studies failed to demonstrate a relationship between dental occlusion and body posture [12, 13]. Recent studies have demonstrated that when comparing the influence of dental occlusion in stable and unstable balance, results revealed a nonsignificant difference in stable conditions but significant differences in unstable balance and fatigue of the subjects [10, 14].

In recent years, research addressing the importance of body posture for athletic performance in pistol shooting [15–17] has suggested that posture is one of the main factors affecting performance in this sport [18]. For instance, it was shown that the movement of centre of pressure determines the movement of the gun-body system, with anterior-posterior body sway accounting for 8% of the variability in horizontal accuracy, and mediolateral body sway accounting for 40% of vertical accuracy variance [18]. Moreover, pistol movement control and a steady upper limb posture were important for shot accuracy [18, 19]. However, other reports found contradictory results, since no connection was reported between body sway and shooting performance [15, 20]. Athletes sometimes sustain the arm for an extended period of time, or perform multiple repeated movements, which can cause an increase in muscle arm stiffness. This has been related to an increase in arm amplitude of motion [21] and can be also relevant for shooting accuracy, since a stable arm is a factor that affects performance [19]. Thus, whether body posture affects performance in pistol shooting remains unknown. In addition, despite the potential ergogenic role of OS in improving balance control and COP for optimal sports performance, to date only one study has explored this question in the context of pistol shooting [9]. Notably, this report shows that OS improves balance control and performance in pistol-shooting athletes. Numerous analyses show that OS has ergogenic effects on body strength, and a positive association between OS and muscle activity in the upper body during isometric tasks has been well established [22–25]. Given that a stable arm is essential for shot accuracy, and that postural tremor during aiming significantly affects performance [19], it is plausible that OS may improve performance in shooting sports by increasing strength in the upper body muscles. In the present study, we examined the acute effects of OS on body sway, upper limb muscle activity, and shot accuracy in healthy 10 m-pistol-shooting athletes.

## 2. Materials and Methods

### 2.1. Subjects

Thirteen national-level male shooters were recruited (age = 38.8 ± 10.9 yrs; weight = 79.7 ± 10.7 kg; height = 1.75 ± 0.1 m) for this study. All athletes had recently been submitted to a physical exam in order to have their shooting license reissued, being deemed fit for sport competition.

Sample size was estimated using effect size [26], so that it could insure a test power of 80.8% and an effect size of 0.710. To achieve these values, a sample size of 21 subjects was required. Unfortunately, this number of subjects was not possible to achieve, due to the low number of high-level athletes available. The inclusion of more athletes could jeopardize the results, since medium- and low-level athletes have more variability in their performance, and it would not be possible to ascertain if changes in results were due to changes in mandibular position or athlete intrinsic variation.

A dental examination performed by an expert dental practitioner confirmed that none of the subjects had a temporomandibular joint (TMJ) disorder or cervical pathology.

For occlusal splint (OS) and placebo splint (PS) fabrication, full maxillary and mandibular arch impressions were taken using an irreversible hydrocoloid (Zhermack OrthoPrint, Rovigo, Italy) and poured in Type III dental stone (Blue Stone, Toledo, Spain). Facebow records were obtained using an arbitrary facebow (Artex FaceBow, Amman Girrbach AG, Koblach, Austria).

Centric relation (CR) was determined after patient deprogramming (cotton rolls interposed between the arches for 4 to 5 minutes) with a Leaf Gauge (Great Lakes Orthodontics, Tonawanda, USA) and subjects were asked to close and slide forward/backward 2 or 3 times and then holding the most posterior comfortable, nonrestrained position, without operator guidance. Centric relation records were obtained with a polyvynilsiloxane bite registration material (VPS-Hydro Bite, Henry Schein, Melville, USA).

Maxillary stone casts were related to an Artex CP semiadjustable articulator (Amman Girrbach AG, Koblach, Austria) with the use of an Artex Transfer Jig (Amman Girrbach AG, Koblach, Austria) and secured in place with the use of mounting plaster (Quick Rock, Protechno, Vilamalla, Spain). Mandibular stone casts were related to the maxillary casts interposing the trimmed centric relation records and secured in place with the use of mounting plaster (Quick Rock, Protechno, Vilamalla, Spain). According to Alexander et al. [27], measurements of articulator mountings are statistically significant for the determination of mandibular positions (maximum intercuspation versus centric relation) and positively correlate to MRI measurements.

**Figure 1:**
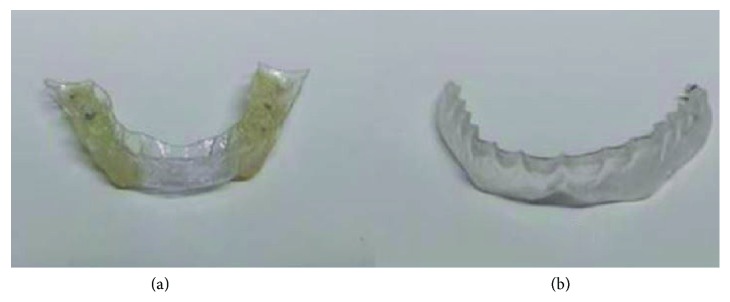
Occlusal appliances used. (a) Occlusal splint; (b) placebo splint.

Using a Vacuum Former machine (Easy Vac, Baekseokdong, South Korea), 1 mm thermoforming foils (Erkodent, Pfalzgrafenweiler, Germany) were adapted over the maxillary casts, trimmed, and adjusted in the articulator at the minimum vertical dimension of occlusion allowed by the thickness of the thermoformed foil, to the requisites of a stabilization splint in a CR position. Splints were tried in the subject's mouth, checked for stability, retention, and comfort and occlusal adjustments were performed until a mutually protected occlusion, coincident with TMJ centric relation, was obtained. PS was obtained following a similar fabrication protocol and trimmed in a way that did not interfere with tooth contact in maximum intercuspation (Figure 1).

The OS repositioned the TMJ in a CR position, which is considered the most stable position for the mandible [5]. The CR is achieved when the TMJ condyles are in their most anterior superior position in the articular fossae, which enables them to be seated in a congruent skeletal arrangement [5].

The repositioning of the TMJ in a CR position was obtained using a modified Lucia jig (anterior flat plane) for deprogramming and registration of a mandibular orthopedically stable position using repeated end point arch of closure. A total of three bite registrations were obtained. A semiadjustable articulator mounting, with an arbitrary face bow, was performed and a split-cast technique was used to validate the interarch relation.

### 2.2. Instrumentation

**Figure 2:**
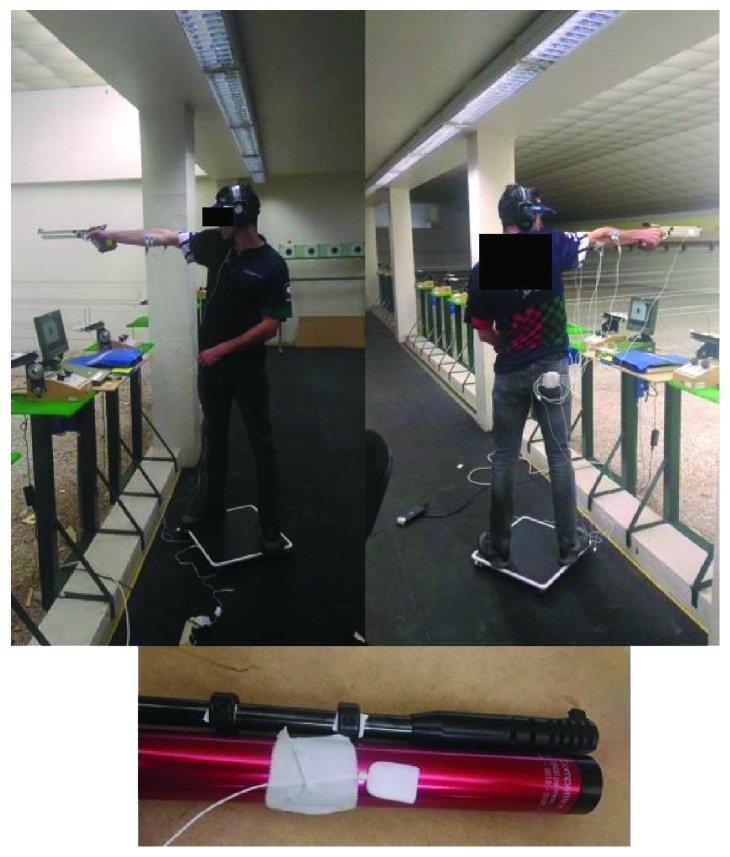
Position of pistol shooters in the platform and position of the accelerometer.

Centre of pressure (COP) position was used to evaluate body sway. COP parameters were quantified using a force platform (Biosignalplux, Lisbon, Portugal) with a dimension of 4500 × 4500 mm. The force platform was positioned directly below the subjects, in the area where they could stand in their favourite position. Muscular activity of the upper limb that holds the pistol was monitored by using surface electromyography (EMG) with bipolar surface electrodes (Biosignalplux, Lisbon, Portugal). The muscles monitored were as follows: medial deltoid, upper trapezius, biceps brachii, flexor digitorum superficialis, and extensor digitorum. The preparation of the skin and electrode placement were described in a previous research [28]. To assess the precise timing of the shot, an accelerometer was used (Biosignalplux, Lisbon, Portugal) and placed on the anterior portion of the gun barrel [19] (Figure 2). The data from the force platform, EMG, and accelerometer were synchronized and recorded simultaneously at 1000 hz with a specific software (OpenSignals, Biosignalplux, Lisbon, Portugal).

Shooting performance was indicated by scores, from 0 to 10.9, consistent with the scoring protocol used in international shooting competitions. For record scoring, an electronic target and control unit were used (SIUS AG, Effretikon, Switzerland).

### 2.3. Experimental Protocol

Testing took place in an indoor national-class competition 10 m-shooting range. Each subject was briefed individually on the testing procedure, and after the experimenter answered every question, the subjects were asked to sign the informed consent form. All subjects used their own personal pistols.

The experimental procedure began with a warm up of 10 shots to allow the subjects to get accustomed to the sensors and to find a stable stance position on the force platform. Next, the subjects performed two series of 10 shots for each condition (OS, PS, and N) in a randomized order. For each experimental condition (OS, PS), no specific indications were given to the subjects on how they should place the mandible, hence the mandibular was always in a rest position. A rest period of three minutes was allowed between series. The total number of shots per condition [20] is in agreement with procedures used in previous studies [15, 18].

### 2.4. Signal Processing and Analysis

**Figure 3:**
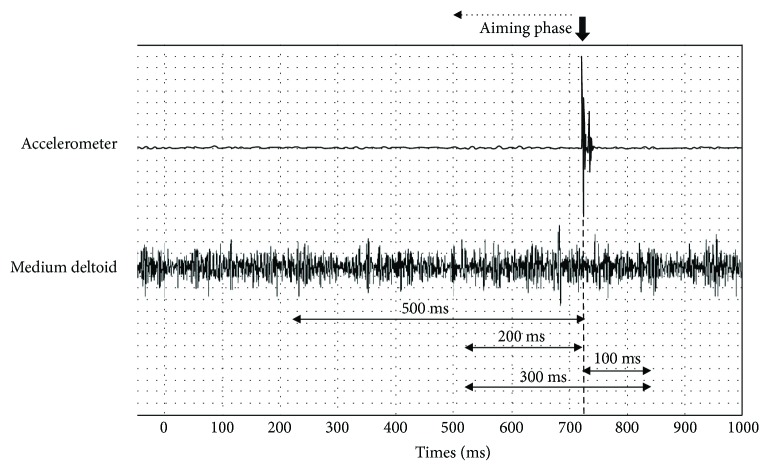
Representative data of accelerometer and EMG signal analysis windows during a shooting task. The onset of the shot was determined by the abrupt change in accelerometer trace.

The EMG signal was amplified with a band-pass filter (25–500 Hz), common-mode rejection ratio (CMRR) of 110 dB, and a gain of 1000. The signal was then digitally filtered (20–500 Hz), rectified, smoothed through a low-pass filter (12 Hz, fourth-order Butterworth digital filter), and amplitude normalized using the peak 100 ms EMG signal on the normal condition of each subject. Normalized average EMG was determined for each muscle, series, and condition using time windows before the shot (500 ms, 200 ms), after the shot (100 ms), and including the shot (300 ms) (Figure 3). The reason for assessing smaller time windows prior to the shot is because it can provide more precise discrimination between different phases, and thus provide additional information on muscle activation and COP parameters that could be masked by using one larger sample window (500 ms).

The signal from the force platform was passed through a digital amplifier with a gain of 400 and processed with a low-pass filter (10 Hz, second-order Butterworth digital filter). The same time windows were used to perform COP parameter analysis. The following COP parameters were obtained: total distance, anteroposterior (AP) distance, medium lateral (ML) distance, AP amplitude oscillation, MP amplitude oscillation, oscillation area, total oscillation velocity, AP oscillation velocity, and ML oscillation velocity. All the processing and analyses of the signals were performed with MATLAB software (version 2013a, MathWorks Inc., Natick, Massachusetts, USA) by using a customized routine.

Mean values and standard deviation for each experimental condition were used to estimate the coefficient of variation of each variable.

### 2.5. Statistical Analysis

Data from all the COP parameters, EMG, and shooting scores were tested for normality using the Shapiro-Wilk test. When the data showed a normal distribution, a repeated-measures ANOVA test was applied. For data with a nonnormal distribution, a Friedman test was used. Significance level was set at 5%. All statistical analyses were processed in IBM SPSS Statistics 22.0 (IBM Corporation, New York, USA).

## 3. Results

The results for the COP parameters and EMG muscle activity measurements are presented in Table 1. No significant differences were found in body sway for any of the conditions, as assessed with COP parameters. Moreover, the EMG measurements revealed no differences in muscular activity between conditions in every muscle analysed. The mean shooting scores for each condition (OS = 9.46 ± 1.06; N = 9.51 ± 0.91, and PS = 9.47 ± 0.99) show that neither OS nor PS affected shooting performance (*p* = 0.212). A coefficient of variation was calculated for the shooting scores (OS = 12.24 ± 1.30; N = 15.37 ± 1.77, and PS = 12.11 ± 1.21) to determine whether there were any changes in shot dispersion. However, no significant differences were detectable between conditions (*p* = 0.069).

## 4. Discussion

Over recent decades, a number of studies reported that changes in occlusion affects strength and body posture [23, 25, 29, 30], and it has been suggested that OS may improve static posture [4, 6, 7] and influence balance recovery [31], possibly by reducing mediolateral sway [8]. Consistent with this, a functional correlation between the trigeminal nerve and the upper limb systems was proposed [32, 33]. Moreover, increased strength [22, 23, 25] and higher EMG muscle activation in the upper limbs have been detected in subjects using OS during isometric tasks [24]. However, other similar studies report conflicting results [34–37]. To address this question, we investigated how OS influences body sway, upper limb muscle activation, and shot accuracy in male pistol-shooting athletes. Our results suggest that changes in occlusion status induced by customised OS do not affect any of these variables. Specifically, no significant changes in a range of body sway parameters were detected in subjects using OS. Shooting performance and muscle activity measured by surface EMG in five upper limb muscles also remained unchanged in the OS condition.

Arm strength, and particularly grip strength, may be essential for pistol shooting because they affect arm stability and/or tremor [19, 38]. Recently, a growing number of studies have assessed the effects of OS on strength and muscle activity in the upper limbs [24, 39, 40] and other body regions [41–43]. Our results contradict two previous reports showing that isometric grip strength and upper body muscle activity are increased in subjects using OS [22, 24]. However, these studies assessed maximal muscle contraction tests, whereas in pistol shooting the upper arm muscles must maintain a steady position for a period of time, which requires a sustained (and not a maximal) isometric muscle contraction. Another study previously investigated whether OS that reposition the TMJ to CR affect body sway in pistol and rifle shooters [9]. In this study, subjects using OS show an improvement in shooting performance and balance control, which contradicts our data. Methodological differences may explain this discrepancy in outcomes. First, in our study all subjects were pistol shooters, whereas Gangloff et al. [9] included both pistol and rifle shooters in their sample. As rifle shooters have smaller body sway areas than pistol shooters [17], this may have skewed the results. Second, the control condition (no splint) in our study was a relaxed position (the subjects' “natural” position), but in Gangloff et al. [9] the subjects were asked to clench their teeth in the control condition.

It is important to note that the large between-individual variability in our results (Table 1) may compromise the detection of an effect. Indeed, except for the 500 ms time window, subjects using OS demonstrated a decrease in oscillation area when compared to the control and PS conditions, but with no statistical significance. These decreases in oscillation areas are similar to a previous study [33]. This methodological limitation could be overcome by using a larger sample.

In pistol shooting, visual cues necessary for maintaining postural control are somewhat compromised because vision is directed at sighting and cannot be preferentially used for postural stability [44]. Thus, during shooting tasks posture control requires additional inputs from other sensory systems, such as proprioception and vestibular signals. Moreover, since shooters are in a bipedal position, the CNS has various degrees of freedom throughout the body to achieve the postural stability and accurate upper arm positioning critical for performance. It is therefore possible that shooting athletes develop specific motor control strategies, mostly based on vestibular and proprioception cues that are less sensitive to new physiological inputs, such as those triggered by changes in dental occlusion induced by OS. Consistent with this, a 4-week longitudinal study revealed that the effects of OS on postural activity were only noticeable a few days after the subjects started wearing the splints [7], and this could explain why we could not detect any significant changes in body sway parameters in our study. Shooting athletes may have a specific motor control strategy for maintaining balance that requires an adaptation period to incorporate physiological changes induced by OS (before producing any noticeable changes in posture).

These strategies may also be influenced by other aspects. The increase in muscle stiffness to reduce the arm tremor amplitude, performed by the subjects in order to maintain a more stable position could actually have an opposite effect, since upper arm muscle stiffness has been related to an increase in arm tremor amplitude [21]. Additionally, the level of muscle fitness of the subjects has also been correlated with muscle tremor. Specifically, subjects that perform resistance training have a chronic reduction in muscle tremor amplitude, which is associated with a reduction in upper limb coactivation (stiffness) [45]. This is a limitation of the present study, since muscle resistance training of each athlete was not assessed in our study.

One other factor that could have played an important role in the outcomes of this study is jaw clenching. It has been demonstrated that clenching has an effect on muscle activity [46], grip strength [47], and posture [2, 11]. Moreover, involuntary clenching of the jaw has been shown to occur in physical activities where strength is involved [48]. Supporting this notion is a study that assessed posture in subjects, while clenching their jaw in the CR position [11]. Results in this study revealed that COP trajectory length was shorter in the CR position, meaning that body posture was more stable. In our study, we did not control the clenching of the jaw, but we recognize that it may have occurred. This is a limitation in our study. Also, this variable is not controlled in many of the studies addressing the relationship between dental occlusion and body posture [2, 4, 8, 12, 13, 29]. This could explain why the literature is inconclusive, alongside with other methodological differences (i.e., occlusal splint versus cotton rolls; different platforms used to measure balance), since it is challenging to distinguish the effects of dental occlusion from clenching.

COP parameters could also be affected by another factor: age. Maintaining a still position requires constant contraction of the lower limb muscles at different levels, to preserve body position. Increases in postural sway has been related to increases in age [49]. Since some of the subjects in our study were older adults, this could also have played a role in the variability of the results.

## 5. Conclusions

This study suggests that body sway, upper limb muscle activity, and shot performance are not affected by dental occlusion status. However, the high between-subject variability in our results could have masked changes caused by OS. Future studies would benefit from a longitudinal design focusing on the medium/long-term effects of using OS on balance sway, muscle activity, and shot performance. Likewise, further research should also focus on jaw clenching and dental occlusion and how the balance between these variables could affect body posture. This research would significantly contribute to our understanding of how TMJ positions influence body postural control and performance outcomes in sports.

## Figures and Tables

**Table 1:** Results of the COP parameters and EMG muscle activity on the three experimental conditions. AP—anterior posterior; ML—medium lateral; OS—occlusal splint; N—normal condition; PS—placebo splint.

Variables (unit)	500 ms				200 ms				100 ms				300 ms			
	OS	N	PS	*P* value	OS	N	PS	*P* value	OS	N	PS	*P* value	OS	N	PS	*P* value
COP parameters																
Total distance (cm)	55.3 ± 24.4	53.5 ± 21.2	55.6 ± 22.2	0.583	35.5 ± 15.9	34.2 ± 13.5	35.6 ± 14.2	0.682	26.5 ± 10.7	26.0 ± 9.9	27.5 ± 10.3	0.583	44.5 ± 18.8	43.2 ± 16.6	45.3 ± 17.3	0.633
AP distance (cm)	33.1 ± 15.8	32.7 ± 15.1	31.9 ± 13.2	0.695	21.3 ± 10.7	21.0 ± 9.9	20.4 ± 8.9	0.754	25.8 ± 7.4	15.9 ± 7.1	16.1 ± 6.4	0.968	26.7 ± 12.8	26.5 ± 12.1	26.3 ± 10.7	0.976
ML distance (cm)	40.6 ± 23.4	38.3 ± 21.4	41.2 ± 25.1	0.394	26 ± 14.9	24.3 ± 13.5	26.9 ± 15.9	0.464	19.6 ± 10.2	18.6 ± 10	20.1 ± 11.7	0.531	32.8 ± 17.8	30.9 ± 16.7	33.2 ± 18.6	0.492
AP amplitude oscillation (cm)	0.7 ± 0.2	0.7 ± 0.2	0.7 ± 0.2	0.538	0.5 ± 0.2	0.4 ± 0.2	0.4 ± 0.1	0.687	0.4 ± 0.4	0.5 ± 0.4	0.6 ± 0.5	0.068	0.7 ± 0.4	0.8 ± 0.4	0.8 ± 0.5	0.199
ML amplitude oscillation (cm)	0.5 ± 0.4	0.5 ± 0.2	0.5 ± 0.2	0.710	0.4 ± 0.3	0.3 ± 0.2	0.3 ± 0.2	0.926	0.5 ± 0.5	0.6 ± 0.6	0.6 ± 0.6	0.368	0.7 ± 0.6	0.8 ± 0.6	0.7 ± 0.6	0.584
Oscillation area (cm^2^)	0.3 ± 0.1	0.5 ± 0.9	0.4 ± 0.5	0.472	0.1 ± 0.8	0.4 ± 0.01	0.3 ± 0.6	0.338	0.2 ± 0.4	0.7 ± 0.9	0.3 ± 0.7	0.472	0.2 ± 0.2	0.4 ± 0.4	0.3 ± 0.7	0.435
Total oscillation velocity (cm/s)	0.05 ± 0.02	0.05 ± 0.02	0.06 ± 0.02	0.583	0.04 ± 0.01	0.03 ± 0.01	0.04 ± 0.01	0.682	0.03 ± 0.01	0.03 ± 0.01	0.03 ± 0.01	0.583	0.04 ± 0.02	0.04 ± 0.02	0.01 ± 0.02	0.589
AP oscillation velocity (cm/s)	0.03 ± 0.02	0.03 ± 0.02	0.03 ± 0.01	0.740	0.02 ± 0.01	0.02 ± 0.001	0.02 ± 0.001	0.754	0.02 ± 0.01	0.02 ± 0.01	0.02 ± 0.01	0.968	0.03 ± 0.01	0.03 ± 0.01	0.03 ± 0.01	0.959
ML oscillation velocity	0.04 ± 0.02	0.04 ± 0.02	0.04 ± 0.02	0.413	0.03 ± 0.01	0.02 ± 0.01	0.03 ± 0.01	0.464	0.02 ± 0.01	0.02 ± 0.01	0.02 ± 0.01	0.531	0.03 ± 0.02	0.03 ± 0.02	0.03 ± 0.02	0.470
EMG (%)																
Medial deltoid	53.0 ± 10.2	53.9 ± 7.5	51 ± 9.8	0.376	50.6 ± 12.2	50.5 ± 10.8	50.9 ± 10.7	0.412	49.7 ± 12.7	48.9 ± 10.8	50.1 ± 11.1	0.663	50.3 ± 11.9	49.5 ± 10.2	50.7 ± 10.2	0.630
Upper trapezius	38.9 ± 16.4	41.8 ± 14.5	39.5 ± 15.6	0.058	38.4 ± 16.4	41.7 ± 14.9	39.1 ± 15.6	0.060	38.6 ± 16.4	41.6 ± 15.2	36.6 ± 16	0.185	38.1 ± 15.9	41.2 ± 14.5	38.6 ± 15.3	0.054
Biceps brachii	31.6 ± 12.4	34.3 ± 11.3	32.9 ± 12.2	0.088	31.7 ± 12.8	34.3 ± 11.7	33.4 ± 12.9	0.197	32.6 ± 12.8	35.3 ± 11.9	34.1 ± 12.8	0.188	37.9 ± 12.6	34.4 ± 11.6	33.5 ± 12.7	0.129
Flexor digitorum	30.2 ± 22.7	28.7 ± 22.7	31.3 ± 22.6	0.666	30.3 ± 22.7	28.6 ± 22.6	31.4 ± 22.6	0.158	31.9 ± 21.6	30.8 ± 21.8	33.4 ± 21.2	0.071	30.4 ± 22.7	28.9 ± 22.6	31.5 ± 22.6	0.657
Extensor digitorum	28.3 ± 12.8	29.0 ± 12.9	28.3 ± 12.6	0.980	28.5 ± 12.3	29.2 ± 13.2	28.46 ± 12.9	0.657	29.9 ± 14.2	28.3 ± 12.8	28.2 ± 12.9	0.08	28.7 ± 13.1	29.1 ± 12.8	27.6 ± 12.6	0.911

## Data Availability

Access to these data will be considered by the authors upon request, by contacting the corresponding author at amandio30@gmail.com.
